# Targeting eIF5A2 inhibits prostate carcinogenesis, migration, invasion and metastasis *in vitro* and *in vivo*

**DOI:** 10.1080/21655979.2020.1774993

**Published:** 2020-06-10

**Authors:** Xiulong Zhong, Hong Xiu, Yanan Bi, Hongmei Zhang, Laizhen Chang, Huifeng Diao

**Affiliations:** a Department of Urology, The Affiliated Hospital of Qingdao University, Qingdao, Shandong, China; b Department of Nursing, The Affiliated Hospital of Qingdao University, Qingdao, Shandong, China; c Department of Medicine, Huaou Group Hospital, Qingdao, Shandong, China

**Keywords:** Prostate cancer, metastasis, eukaryotic initiation factor- 5A2

## Abstract

Overexpression of eukaryotic initiation factor- 5A2 (eIF5A2) has been implicated in promoting tumor cell migration and invasion in many cancers. However, whether eIF5A2 could be as the target for prostate cancer (PCa) treatment is still unknown. In this study, small interfering RNA specific for eIF5A2 (eIF5A2 siRNA) and lentivector for eIF5A2 shRNA (Lv-eIF5A2 shRNA) was performed to down-regulate eIF5A2 expression in PCa PC-3 M IE8 cells and in animal tumor model, respectively. The biological function of eIF5A2 siRNA or Lv-eIF5A2 shRNA on PC-3 M IE8 cell growth, apoptosis, migration, invasion and lung metastasis were explored. The results showed that targeting eIF5A2 inhibited PC-3 M IE8 cell invasion, migration, proliferation and increased cell apoptosis *in vitro*, and inhibited tumor growth and lung metastasis *in vivo*. Analysis of eIF5A2 signaling pathways in the clonal derivatives showed a decrease in MMP-2 and MMP-9 activation and increase in bcl-2 expression. We therefore concluded that therapies targeting the eIF5A2 signaling pathway may be more effective to prevent organ metastasis and primary tumor formation.

## Introduction

The development of prostate cancer (PCa) is initially driven by androgen steroid hormones via the androgen receptor (AR) transcription factor. The first line treatment for prostate cancer that is no longer organ confined is androgen deprivation therapy (ADT) [1]. However, after 2–3 years many patients develop castrate resistant prostate cancer (CRPC) for which treatment options are limited and prognosis is poor [2], meaning there is an urgent need to develop new treatments for advanced prostate cancer.

The eukaryotic initiation factor −5A2 (eIF5-A2) gene, located on chromosome 3q26, was first discovered in the primary ovarian cancer cell line in 2001 and has been classified as an oncogene [3]. Accumulative clinical and experimental, evidence show that EIF5A2 is overexpressed in many malignant tumor tissues, and upregulation of eIF5A2 is associated with poor survival, advanced disease stage, poor response for chemotherapeutic drugs as well as metastasis for patients with cancer [4–10], suggesting that eIF5A2 might be a potential prognostic biomarker for malignancies.

Numerous evidence demonstrated that enforced eIF5A2 enhances cancer cell growth, increases cancer cell metastasis, and promotes chemotherapy resistance through multiple means [4,11–13]. Furthermore, targeting eIF5A2 attenuats tumor growth and metastasis as well as overcomes chemotherapeutic resistance [12–15], suggesting that targeting eIF5A2 may provide an effective approach for the treatment of malignancies.

Liu et al. [16] reported that eIF5A2 was overexpressed in PCa tissues, and enhanced eIF5A2 expression was related with higher tumor stage, recurrence and short survival, indicating that eIF5A2 expression could be as a candidate biomarker for prognosis assessment in prostate cancer. In upper tract urothelial carcinoma, EIF5A2 overexpression was also with intravesical recurrence and poor prognosis after surgery [17,18]. However, whether EIF5A2 could be as the target for PCa treatment is still unknown.

RNA interference (RNAi), mediated by small interfering RNA (siRNA), is effective in selectively inhibiting the expression of specific genes. In the present study, we undertook studies *in vitro* using PC-3 M IE8 cell lines and *in vivo* using a human xenograft PC-3 M IE8 tumor animal model by RNA interference. Mechanisms that lead to function of targeting EIF5A2 were also highlighted. The results of these studies indicate that targeting eIF5A2 in the metastatic PCa cells decreased the migration and invasion *in vitro* and decreased the number of lung metastasis in an orthotopic model *in vivo*.

## Materials and methods

### Cell culture and agents

The human metastatic human PCa cell line PC-3 M IE8 was purchased from ECACC (Shanghai, China). It was cultured in RPMI-1640+10% FBS+1% PS at 37°C in a humidified atmosphere containing 5% CO_2_. The antibodies used were anti- EIF5A2, anti-MMP-2/9, anti-GAPDH and secondary antibody. All the antibodies were purchased from Cell Signaling Technology (Shanghai, China).

### Transient siRNA transfection

siRNA against eIF5A2 was designed and chemically synthesized (Shanghai GenePharma Co., Shanghai, China). Additionally, the control siRNA-NC was also synthesized as a negative control. Both siRNAs were transiently transfected into the PC-3 M IE8 cells using Lipofectamine^TM^ 2000 Transfection Reagent (Invitrogen) according to the manufacturer’s instruction.

### Production of Lentivirus

A shRNA sequence targeting EIF5A2 (EIF5A2 shRNA) was cloned into pWPXL replacing the GFP marker. All recombinant lentiviruses were produced by transient transfection of 293 T cells according to standard protocols. Briefly, subconfluent 293 T cells were cotransfected with 20 ug of a plasmid vector, 15 ug psPAX2, and 5 ug pMD2.G by calcium phosphate precipitation. After 16 h, medium was changed, and recombinant lentivirus vectors were harvested 48 h later and filtered through 0.45 um filters. Virus titers were calculated at 72 h after virus infection by counting the number of GFP-expressing foci divided by the dilution factor. For virus infection, culture cells were incubated with culture medium-diluted virus supernatant supplemented with polybrene (8 ug/mL) for 6 h. To achieve >95% infection efficiency, virus titers of 20 to 40 transduction unit/cells were used.

### Western blot assay

Cells were harvested and solubilized in RIPA buffer, and the whole cell lysates were prepared. Standard Western blotting was carried out using whole-cell protein lysates. The cell lysates resolved on SDS-PAGE gels and transferred to PVDF membranes. Primary antibodies were incubated overnight at 4°C. The antibodies used in the analysis were anti-eIF5A2 and anti-GAPDH. The proteins were detected using HRP-labeled secondary antibodies and visualized using the Amersham ECL System and detection system analyzed (ChemiDoc Touch, BioRad).

### Quantitative real-time RT-PCR

Quantitative PCR was done using a SYBR Green detection system from Bio-Rad. Cells were seeded in a six-well plate and allowed to grow to confluency. Cells were transfected using Lipofectamine 2000 (Invitrogen) and then total RNA was isolated after 72 h. Total RNA was isolated using TRIzol reagent (Invitrogen) per the manufacturer’s instructions. Total RNA was then reverse transcribed into cDNA using Bio-Rad iScript cDNA synthesis kit. Quantitative PCR was last done using the Bio-Rad SYBR green supermix and iCycler detection system.

The primer pairs for qPCR were eIF5A2, bcl-2, MMP-2 and MMP-9.

### Cell viability assays

Equal numbers of human PC-3 M IE8 cells were plated in triplicate in 96-well culture plates and stained with MTT [3-(4,5-dimethylthiazol-2-yl)-2, 5-diphenyltetrazolium bromide] after EIF5A2 siRNA or control siRNA transfection for 72 h. Six replicates were prepared for transfected and cultured until 5 days. After the addition of 200 μl DMSO (dymethylsulfoxide) in each well, the samples were incubated in the dark for 30 min, and then swirled for mixing. Absorbance A at 490 nm was measured using enzymatic reader. Experiments were repeated three times.

### Annexin V-FITC-PI apoptosis detection assay

PC-3 M IE8 cells were transfected with EIF5A2 siRNA or control siRNA for 72 h. Then the cells were washed twice in ice-cold PBS and suspended in ice-cold binding buffer. To the cell suspensions were added 3ul of the annexin conjugate ApopNexin-FITC and 2 ul of 100 ul PI, and the mixtures were incubated for 15 min at room temperature in the dark. Double-staining cells were analyzed with a ﬂuorescence microscope.

### Wound-healing assay

For wound-healing assays, 3 × 10^5^ stable EIF5A2 shRNA or control shRNA transfected PC-3 M IE8 cells were seeded into 6-well plates for 24 h. A scratch was drawn across the center of the well using a plastic pipette tip to produce a sharp, 1-mm-wide wound area. The cells were washed with fresh, FBS-free medium 3 times and incubated in medium containing 1% FBS for 48 h. Cell movement into the wound area was examined using a phase-contrast microscope.

### Cell migration and invasion assay

Cell migration and invasion assays were performed using transwell plates (8-μm pore size, Corning Costar Corp.) and Matrigel (BD). Briefly, PC-3 M IE8 cells were transfected with IF5A2 siRNA or control siRNA for 24 h. Then the cells were resuspended at a density of 1 × 10^5^ cells/ml. BD Falcon Cell Culture Inserts (San Jose, CA, USA) were coated with 50 μL of Matrigel and placed in each well. Plate wells were filled with 600 μL of complete medium, and the upper insert were filled with 100 μL of the cell suspension. Cells that migrated to the bottom side of the membrane were counted using a microscope, and relative cell motility activity was calculated using the following formula: relative cell motility activity = number of migrated or invaded cells from different treatment groups ÷ number of migrated or invaded cells from the control group.

### Experimental grow and metastasis in vivo

The stable EIF5A2 shRNA or CN shRNA transfected PC-3 M IE8 cells were washed and resuspended in PBS. Subsequently, a single-cell suspension containing 106 cells in 0.1 mL PBS was injected into the right flank of immunodeficient mice subcutaneously. The tumor volume was calculated using: tumor volume (mm^3^) = π/6 × a × b^2^. The animals were observed for 35 days after the last injection. After 35 days, tumor xenografts were harvested and analyzed. Lung was examined for metastasis formation. The lungs were removed, weighed, and fixed in 10% formalin. The number of lung tumor colonies was counted under a dissecting microscope. All animal experiments were done in accordance with the animal guidelines at the affiliated hospital of Qingdao University.

### Immunohistochemical examination

Primary tumors excised from mouse xenografts were fixed in formalin. Tissue sections (7 μm) were prepared and stained with anti- EIF5A2, anti-bcl-2, anti-Ki67 and anti-MMP-2/9 (Cell Signaling Technology) according to the manufacturer’s instructions.

### TUNEL Staining

Fresh-frozen sections of tumor tissues from therapy experiments (EIF5A2 shRNA model) were stained by terminal deoxynucleotidyl transferase-mediated deoxyuridine triphosphate nick-end labeling (TUNEL) as the manufacture’s instruction. To quantify apoptotic cells, TUNEL-positive cells were calculated in 10 random fields at 200× from five separate slides per group. Average values are presented.

### Statistical analysis

Quantitative data were presented as the mean±SD of at least three independent experiments. Statistical analysis of data was carried out by Student’s t-test or by one-way ANOVA using Dunnett’s test in multiple comparisons of means. Differences were considered statistically signiﬁcant if the P-value was <0.05

## Results

### Targeting EIF5A2 inhibits proliferation and induces apoptotis in PC-3 M IE8 cells *in vitro*

**Figure 1.**
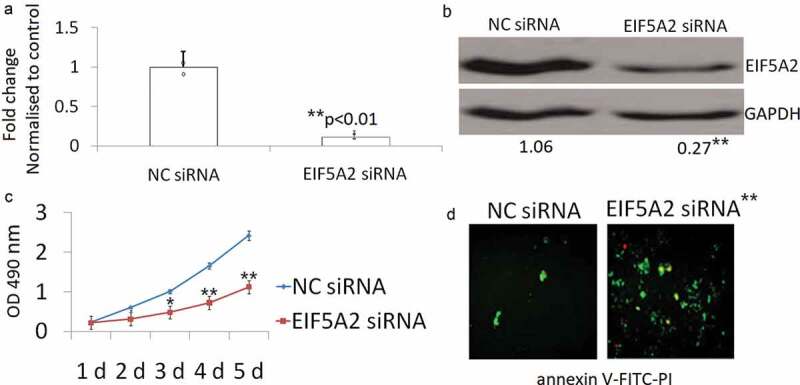
EIF5A2 sliencing inhibits proliferation and induces apoptotis in vitro. a, PC-3 M IE8 cells were transfected with the EIF5A2 siRNA for 72 h. EIF5A2 mRNA was detected by qPCR. b, PC-3 M IE8 cells were transfected with the EIF5A2 siRNA for 72 h. EIF5A2 protein expression was detected by western blot assay. c, Cell proliferation was detected by MTT assay after siRNA transfection for 1–5 days. d, PC-3 M IE8 cells were transfected with the EIF5A2 siRNA for 72 h, then the cells were subjected to annexin V-FITC-PI for apoptosis detect.

PC-3 M IE8 cells were transfected with the EIF5A2 siRNA plasmid for 72 h. As shown in Figure 1(a), EIF5A2 mRNA levels were decreased > 90% in the PC-3 M IE8/EIF5A2 siRNA clone when compared with the NC siRNA transfected PC-3 M IE8/NC siRNA clone. Western blot assay has the similar results as qPCR (Figure 1(b)). Next, the effects of EIF5A2 silencing on both cell proliferation and apoptosis were determined *in vitro*. Analysis of cell proliferation using the MTT assay showed significantly decreased proliferation in the EIF5A2 siRNA clone when compared with the PC-3 M IE8/NC siRNA clone after 3 days transfection (P < 0.05) and 4–5 days transfection (P < 0.01) (Figure 1(c)). Apoptosis of the cell clone was determined using annexin V-FITC-PI staining. EIF5A2 siRNA clone showed 42.4% apoptosis, whareas PC-3 M IE8/NC siRNA clone showed 3.8% apoptosis after 3 days transfection (P < 0.01) (Figure 1(d)).

### Targeting EIF5A2 inhibits migratory potential of PC-3 M IE8 cells *in vitro*

**Figure 2.**
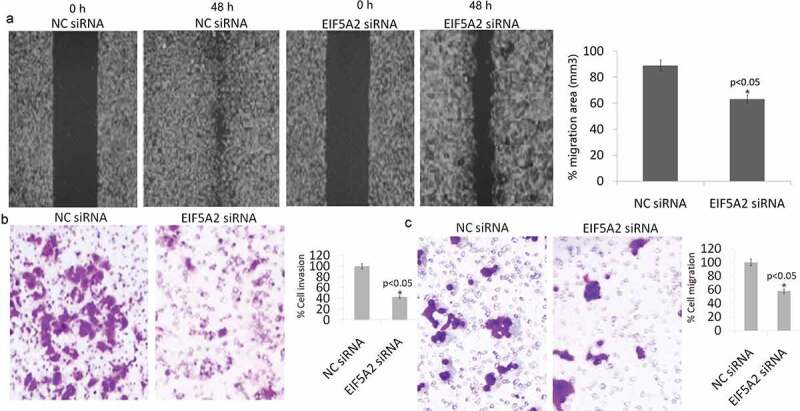
Silencing of EIF5A2 affects migration and invasion of PC-3 M IE8 cells *in vitro*. a,PC-3 M IE8 cells containing EIF5A2 siRNA or NC siRNA were analyzed for cell migration using the wound-healing scratch assay. Cells were ‘wounded’ and monitored for 48 h to determine the rate of migration into the scratched area. b, Invasiveness of cells was determined using a Matrigel invasion; c, migration of cells was determined using transwell migration assay.

PC-3 M IE8 is a highly metastatic PCa cell line. *In vitro* studies were done to determine the effects of EIF5A2 silencing on both migration and invasion of these cell clones. Cell migration was first determined using a wound-healing assay in which cells were scratched and allowed to migrate into the wound area. The amount of migration or wound closure was enumerated 48 h after disruption. The PC-3 M IE8/NC siRNA cells showed 90% wound closure by 72 h, the PC-3 M IE8/EIF5A2 siRNA colone showed 63% wound closure in the same period (Figure 2(a)). Using a Boyden chamber, we determined changes in cell invasiveness after 24 h. Cells were fixed and stained with crystal violet to determine the number of cells that invaded across the membrane. Compared with PC-3 M IE8/NC siRNA cells, the PC-3 M IE8/NC siRNA cells showed more than 60% decrease in invasion (Figure 2(b)) and 55% decrease in migration (Figure 2(c)).

### Targeting EIF5A2 inhibits MMP-2/9 and increases bcl-2 expression

**Figure 3.**
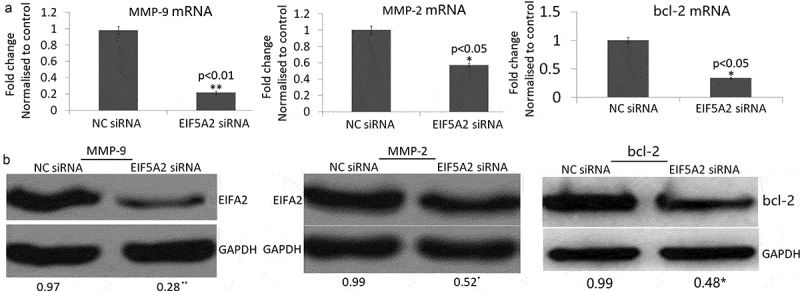
Silencing of EIF5A2 inhibits MMP-2/9 expression in PC-3 M IE8 cells *in vitro*. PC-3 M IE8 cells were transfected with the EIF5A2 siRNA plasmid for 72 h. a, MMP-2 and MMP-9 mRNA expression by qPCR assay; b, MMP-2 and MMP-9 protein expression by Western blot assay.

As shown in Figure 3(a), MMP-2 and MMP-9 mRNA expression was significantly decreased and bcl-2 was significantly increased in the EIF5A2 siRNA colony compared with the NC siRNA colony by qPCR assay. MMP-2 and MMP-9 protein expression was also significantly decreased and bcl-2 was significantly increased in the EIF5A2 siRNA colony compared with the NC siRNA colony by western blot assay (Figure 3(b)).

### Targeting EIF5A2 inhibits tumor growth *in vivo*

**Figure 4.**
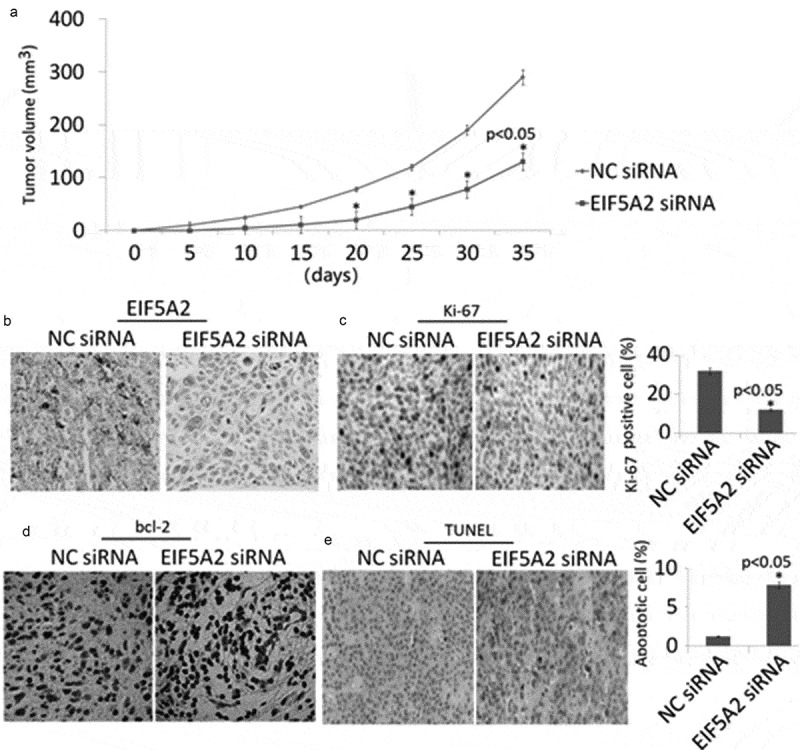
Silencing of EIF5A2 inhibits in vivo tumor growth. a, Effect of injection of EIF5A2 shRNA/PC-3 M IE8 cells or NC shRNA/PC-3 M IE8 cells on *in vivo* tumor growth. Silencing of EIF5A2 resulted in significant inhibition of tumor growth in PC-3 M IE8 cells. b,c,d,e, Representative IHC staining c of EIF5A2, Ki67, bcl-2 and TUNEL staining in parafﬁn-embedded specimens from the subcutaneous tumors.

To determine the role of targeting EIF5A2 on PC-3 M IE8 tumor growth *in vivo*, the stable EIF5A2 shRNA or CN shRNA transfected PC-3 M IE8 cells were subcutaneously injected into the right flank of nude mice and observed its growth for 35 days. As shown in Figure 4(a), EIF5A2 downexpression significantly inhibited xenografted tumor growth compared with vector controls. Immunohistochemical analysis showed that EIF5A2 and Ki67 expression was decreased (Figure 4(b-d)), and TUNEL positive cells (Figure 4(e)) was increased in EIF5A2-downxpressing tumors, indicating that targeting EIF5A2 inhibited tumor growth by inhibiting cell proliferation and increasing cell apoptosis, respectively.

### Targeting EIF5A2 inhibits lung metastasis *in vivo*

**Figure 5.**
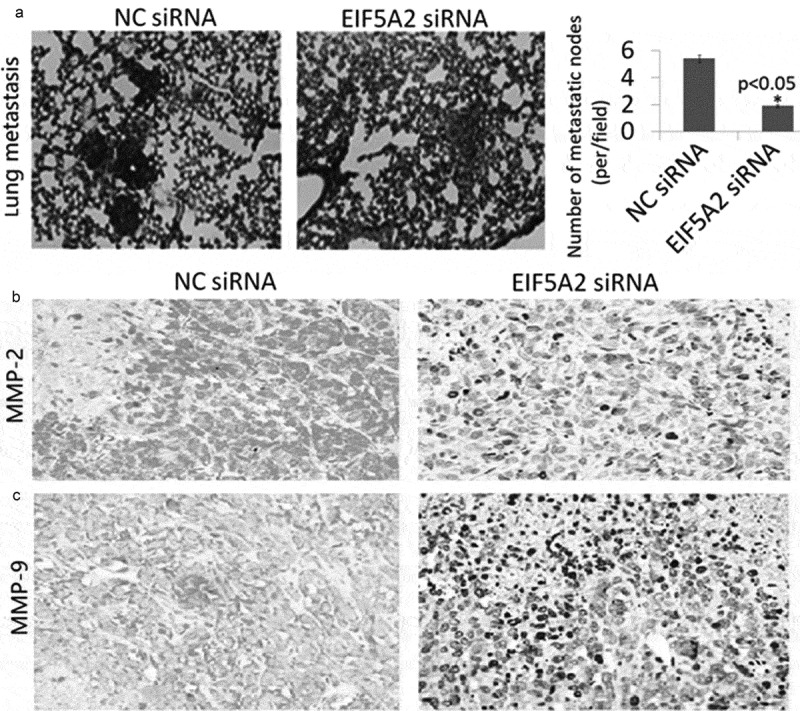
Silencing of EIF5A2 inhibits PC-3 M IE8 cells lung metastasis in vivo. a, Lung a metastasis mouse models were used to analyze the effect of silencing of EIF5A2 on tumor metastasis in vivo. Representative images of hematoxylin- and eosin-stained sections of lung of the mice from the indicated groups. The number of lung metastatic foci revealed by hematoxylin and eosin staining is calculated microscopically, represented as the mean ± SEM. b,c, Representative IHC staining of MMP-2 and MMP-9 staining in parafﬁn-embedded specimens from the subcutaneous tumors.

We confirmed the in vitro results in metastatic tumor models of PC-3 M IE8 cells, finding that EIF5A2 sliencing markedly decreased lung metastasis of PC-3 M IE8 cells (1.2 ± 0.4) compared to the CN shRNA transfected PC-3 M IE8 cells (3.8 ± 1.2) (P < 0.05, Figure 5(a)). Immunohistochemical analysis showed that MMP-2/9 expression was decreased in the EIF5A2-downxpressing tumors compared with the CN shRNA transfected colony (Figure 5(b-c)).

## Discussion

The eukaryotic translation factor 5A (eIF5A) originally identified as an initiation factor, was later shown to promote translation elongation of iterated proline sequences [19]. Two eIF5A isoforms eIF5A1 and eIF5A2 are 84% identical. However, the biological functions of these two isoforms may be significantly different [20]. Recently, it has demonstrated that eIF5A is widely involved in the pathogenesis of many diseases, including upper urinary tract urothelial carcinoma and prostate cancer (PCa) [16–18]. In particular, eIF5A2 plays an important role in regulating tumor growth, invasion and metastasis [4,13,21,22]. It was also shown to serve as a potential biomarker and target for the diagnosis and treatment of cancers [5,6,8].

Results of the present study indicated that silencing of eIF5A2 inhibits PC-3 M IE8 cell invasion and migration *in vitro* and lung metastasis *in vivo*. Furthermore, silencing of eIF5A2 also induces PC-3 M IE8 cell apoptosis and inhibits cell proliferation *in vitro* and *in vivo*, and tumor grow *in vivo*. Therefore, targeting the eIF5A2 signaling may be more effective to prevent organ metastasis and primary tumor formation.

Matrix metalloproteinases (MMPs) are a family of zinc-dependent proteolytic enzymes that degrade various components of extracellular matrix (ECM) [23]. Among the MMPs, MMP-2 (gelatinase A) and MMP-9 (gelatinase B) have been widely studied that degrade both gelatins and collagens of ECM [24]. In cancer pathology, MMP-2 and MMP-9 are mainly implicated in the formation of new blood vessels through angiogenesis; MMP-2 and MMP-9 facilitate the migration of tumor cells to blood vessels by the degradation of basement membrane ECM proteins [25]. Previous study has demonstrated that both melanoma cell invasion and matrix metalloproteinase 2 (MMP-2) activity increased and decreased with EIF5A2 overexpression and knockdown, respectively [26]. In addition, ablation of endogenous EIF5A2 inhibited tumor angiogenesis by reducing MMP-2 expression [27]. Western blots and qPCR for MMP-2 and MMP-9 in the present study indicated a 80–90% decrease in mRNA and protein expression in MMP-9, and 40–50% decrease in mRNA and protein expression in MMP-2. This data suggested that EIF5A2 might mainly function via MMP-9.

Initial studies to characterize the clonal derivatives of PC-3 M IE8 cells constitutively expressing EIF5A2 siRNA showed significan changes in apoptosis, indicating that changes in the number of metastasis are partly due to increased apoptosis. Cell proliferation analysis indicated a 50–60% decrease in proliferation in vitro. This decrease in proliferation in response to EIF5A2 silencing seems to be due to an increase in bcl-2 expression.

## Conclusion

The present study suggests that therapies targeting EIF5A2 in prostate cancer (PCa) may be effective in decreasing metastasis and inhibiting tumor grow. Therefore, EIF5A2 may be a potential therapeutic target for PCa.

## Highlights

1. Targeting eIF5A2 inhibits invasion, proliferation and induces apoptosis in PC-3M IE8 cells *in vitro*
2. Targeting eIF5A2 inhibits tumour growth and lung metastasis in PC-3M IE8 cells *in vivo*
3. Targeting eIF5A2 regulates MMP-2/9 and bcl-2 pathway to play an anti-tumor effect in *in vitro* and *in vivo*
